# Supplementary material for: Multi‐dataset identification of innovative feature genes and molecular mechanisms in keratoconus
Source: J Cell Mol Med. 2024 Sep 19;28(18):e70079. doi: 10.1111/jcmm.70079 (PMC11412914; doi:10.1111/jcmm.70079)
Supplement: Supplementary file 2 — Material S2. [file JCMM-28-e70079-s002.zip › 7.GSEA analysis/2.ATOH7/KEGG_BIOSYNTHESIS_OF_UNSATURATED_FATTY_ACIDS.html]

Details for gene set KEGG\_BIOSYNTHESIS\_OF\_UNSATURATED\_FATTY\_ACIDS[GSEA]

|  || Dataset | ATOH7.group.cls#h\_versus\_l |
| Phenotype | group.cls#h\_versus\_l |
| Upregulated in class | l |
| GeneSet | KEGG\_BIOSYNTHESIS\_OF\_UNSATURATED\_FATTY\_ACIDS |
| Enrichment Score (ES) | -0.16851352 |
| Normalized Enrichment Score (NES) | -0.56213194 |
| Nominal p-value | 0.9749518 |
| FDR q-value | 1.0 |
| FWER p-Value | 1.0 |
Table: GSEA Results Summary

  

Fig 1: Enrichment plot: KEGG\_BIOSYNTHESIS\_OF\_UNSATURATED\_FATTY\_ACIDS      
 Profile of the Running ES Score & Positions of GeneSet Members on the Rank Ordered List

  

| SYMBOL | TITLE | RANK IN GENE LIST | RANK METRIC SCORE | RUNNING ES | CORE ENRICHMENT || 1 | ELOVL5 | na | 2046 | 0.341 | 0.1408 | Yes |
| 2 | ELOVL2 | na | 8278 | 0.113 | 0.0275 | Yes |
| 3 | YOD1 | na | 8518 | 0.108 | 0.0839 | Yes |
| 4 | PECR | na | 10505 | 0.073 | 0.0698 | Yes |
| 5 | ACOT7 | na | 11091 | 0.065 | 0.0909 | Yes |
| 6 | ACOT4 | na | 12160 | 0.051 | 0.0897 | Yes |
| 7 | ACOT1 | na | 14919 | 0.022 | 0.0231 | Yes |
| 8 | HADHA | na | 16925 | 0.005 | -0.0316 | Yes |
| 9 | TECR | na | 18199 | -0.006 | -0.0650 | Yes |
| 10 | FADS1 | na | 21794 | -0.041 | -0.1447 | Yes |
| 11 | ACOX3 | na | 21920 | -0.042 | -0.1237 | Yes |
| 12 | ELOVL6 | na | 23416 | -0.061 | -0.1311 | Yes |
| 13 | SCD | na | 24053 | -0.070 | -0.1081 | Yes |
| 14 | HSD17B12 | na | 24767 | -0.082 | -0.0808 | Yes |
| 15 | BAAT | na | 25243 | -0.090 | -0.0420 | Yes |
| 16 | ACOX1 | na | 25688 | -0.098 | 0.0027 | Yes |
| 17 | ACAA1 | na | 26532 | -0.115 | 0.0461 | Yes |
| 18 | SCD5 | na | 27173 | -0.130 | 0.1038 | Yes |
| 19 | FADS2 | na | 29483 | -0.194 | 0.1511 | Yes |
Table: GSEA details [plain text format]

  

Fig 2: KEGG\_BIOSYNTHESIS\_OF\_UNSATURATED\_FATTY\_ACIDS      
 Blue-Pink O' Gram in the Space of the Analyzed GeneSet

  

Fig 3: KEGG\_BIOSYNTHESIS\_OF\_UNSATURATED\_FATTY\_ACIDS: Random ES distribution      
 Gene set null distribution of ES for **KEGG\_BIOSYNTHESIS\_OF\_UNSATURATED\_FATTY\_ACIDS**
